# Concrete Experiences and Support Needs Regarding the Euthanasia Practice in Adults With Psychiatric Conditions: A Qualitative Interview Study Among Healthcare Professionals and Volunteers in Belgium

**DOI:** 10.3389/fpsyt.2022.859745

**Published:** 2022-03-11

**Authors:** Monica Verhofstadt, Kurt Audenaert, Freddy Mortier, Luc Deliens, Axel Liégeois, Koen Pardon, Kenneth Chambaere

**Affiliations:** ^1^End-of-life Care Research Group, Vrije Universiteit Brussel (VUB) & Ghent University, Brussels, Belgium; ^2^Department of Psychiatry, Ghent University Hospital, Ghent, Belgium; ^3^Bioethics Institute Ghent, Ghent University, Ghent, Belgium; ^4^Faculty of Theology and Religious Studies, KU Leuven, Louvain, Belgium; ^5^Organisation Brothers of Charity, Ghent, Belgium

**Keywords:** euthanasia, mental disorder, end-of-life decisions, assisted suicide, adult psychiatry

## Abstract

**Objective:**

Although euthanasia in the context of adult psychiatry is legalized in Belgium, it poses major ethical and clinical challenges for the health care professionals and volunteers involved. This study aimed to address these members' concrete experiences and support needs.

**Methods:**

A qualitative semi-structured interview study was conducted with 16 physicians and 14 other health care professionals and volunteers, with at least one concrete experience with euthanasia requests and procedures concerning adults with psychiatric conditions.

**Findings:**

Concrete experiences concerned the following 8 domains: (1) the impact of euthanasia on the clinical trajectory and (2) on the therapeutic relationship, (3) internal and (4) external collaborative partnerships, (5) patients' social inner circle (non-)involvement, (6) the use of recently published guidelines and, (7) the first criminal trials on this topic, and (8) the act of euthanasia. The following 8 main support needs emerged; (1) protocols addressing specific sub-populations and pathologies, (2) protocols specifically drawn up for non-medics, (3) guidance on how to adequately implement the two-track approach, (4) (after)care for patients, (5) (after)care for the health care team, (6) guidance on the patient's social inner circle involvement, (7) enhanced education measures, and (8) enhanced financial measures, including incentives for holistic, palliative care approaches.

**Conclusion:**

The health care professionals and volunteers reported many positive and negative experiences in dealing with euthanasia requests in adult psychiatry. They reported several support needs across the extensive euthanasia trajectory, pertaining to concrete management of thorny issues that guidelines do not (yet) touch on. Important implications of our study relate to tackling these existing issues, and to paying sufficient attention to the impact of a euthanasia trajectory on all actors, including the patients and their social inner circle, involved.

## Introduction

Medical assistance in dying, defined as the act to end life by providing, prescribing or administering lethal medication at the competent patient's explicit request, is—under certain conditions—legal in an increasing number of countries around the globe (1). “Euthanasia” refers to the act of a physician administering the lethal medication; “physician-assisted suicide” refers to the act of prescribing or providing the medication to the patient, who then self-administers it.

Belgium (2) is one of the countries—next to the Netherlands (3), Luxembourg (4), Spain (5), Germany (6) and Switzerland (1) —that does not exclude adults who suffer predominantly from irremediable psychiatric conditions from medical assistance in dying per definition. Canada considers to expand current legislation to this specific patient group in 2023 (7).

The Belgian central requirements include having the necessary mental competence to express a voluntary, well-considered and repeated euthanasia request, experiencing unbearable suffering that cannot be alleviated and that is based on an incurable medical condition (see Box 1 in OSF for all legal criteria). Although adults with psychiatric conditions can be potentially eligible for euthanasia (i.e., the act of a physician administering the lethal medication to a patient), it remains a highly controversial and extremely complex end-of-life practice in terms of whether and when these patients can meet all legal criteria (8). Apart from the difficulties in assessing the legal criteria, these euthanasia assessment procedures are also professionally and emotionally demanding. For instance, physicians have to deal with a higher level of uncertainty in psychiatry (in terms of diagnostics, prognosis, treatment efficacies, and outcome of psychiatric conditions) and the tension between suicide prevention and euthanasia (8, 9). In addition, euthanasia assessment procedures concerning this patient group, may take an emotional toll, as a recent survey study revealed that physicians may feel e.g., pressured by the patient, the patient's social inner circle, colleague-physicians and/or the affiliated institution to (dis)approve the euthanasia request (10).

Recent empirical evidence revealed that although three-quarters of psychiatrists in Belgium are in favor of euthanasia legislation that does not exclude this specific patient group, only a minority is willing to actively engage in their own patient's euthanasia procedure (39%), to be engaged as advising physician (30%) or performing physician (<10%) (11). The reluctancemay be reflected by the decrease in the number of performed euthanasia cases that were reported to the Federal Evaluation and Control Committee on Euthanasia: from 43 adult patients with psychiatric conditions that died by euthanasia in the year 2015 (12) to 23 patients in 2019 (13).

The growing reluctance among physicians may be ascribed to a recent court case in 2020 (see Box 2), in which three physicians stood trial for potential offenses against the euthanasia law concerning one adult with psychiatric conditions. But even before the court case, it was clear that physicians needed more support in the handling of euthanasia request based on psychiatric reasons. In the years 2017–2019, several guidelines were published and recommended more strict criteria than required by Law, e.g., the consultation of not one but 2 psychiatrists, the need for 2 positive advices instead of 2 advices of which the outcome is not legally binding, and the need to explore all reasonable alternatives to death, also from a non-medical, psychosocial perspective (14). Although these additional recommendations are not legally binding, many physicians are confronted with increased stringency and increased awareness of being prone to court cases. And although these guidelines may offer useful guidance for enhancing clinical euthanasia management in psychiatry, differences in approaches remain, and not all existing bottlenecks have been identified, let alone addressed adequately.

Box 2 **Milestones in the euthanasia practice in Belgium: (1) the establishment of end-of-life information and consultation centres, (2) the establishment of a rehabilitation center and action group, (3) the guidelines on the management of euthanasia in the context of psychiatry, and (4) the court cases based on potential unlawfully practised euthanasia in adult patients suffering from psychiatric conditions.**

**End-of-life information and consultation centres**

**I) Recht op Waardig Sterven (Right to Die with Dignity)**

In the first half of the 1980s, Right to Die Organisations were founded in the Flemish and French-speaking part Belgium, namely *Recht op Waardig Sterven* (RWS) and *L'Association pour le Droit de Mourir dans la Dignité* (Association for the Right to Die with Dignity). Their activism resulted in several legislative proposals on euthanasia legislation from 1984 onwards. Since euthanasia enactment, their activism relates to e.g., informing individuals on the medical end-of-life options in Belgium and support them with the administrative paperwork surrounding (some of) these options.

**II) LEIF (Life End Information Centre)**

The Right to Die Organisation RWS founded the Flemish organisation Life End Information Forum (LEIF) in 2003. LEIF provides e.g., training for physicians and nurses to increase their knowledge on end-of-life legislation and how to implement it in practice and training for physicians to act as advising or performing physician. LEIF developed and published guidelines on how to handle euthanasia requests and to perform euthanasia.

**III) End-of-life consultation centres**

LEIF established three end-of-life consultation centres (ULteam in 2011, LEIF Western-Flanders in 2013 and LEIF. Ghent in 2015) with the aim to effectively engage in euthanasia assessment procedures, especially for those patients confronted with a neglected euthanasia request. These consultation centres consist of an interdisciplinary team of physicians, psychologists (psychiatric), nurses, ethicists, and legal experts, with extensive expertise in the management of complex euthanasia cases.

Due to dissension on how to handle these cases, LEIF. Ghent has been deposed as regional LEIF centre and follows its own course as “End-of-Life Questions Ghent” (publicly known as Vonkel) since 2017.

**Other initiatives**

**I) REAKIRO**

Reakiro is a place in Louvan (2020) and West-Flanders (as of 2022) where all individuals considering euthanasia on grounds of unbearable psychological suffering, and their relatives, can go to. The primary focus of Reakiro is rooted in the rehabilitation approach, characterised by an active orientation toward life, toward (re)discovering meaning, purpose and hope in life, *without* excluding the option of euthanasia. This rehabilitation approach is founded on the following 4 main pillars to qualitative (end-of-life) care: the medical, psychological, social and existential care approach.

**II) REBEL**

REBEL is an activist group that consists of Belgian clinicians and academics of all disciplines and philosophies who express their concerns regarding the current euthanasia law and the euthanasia practice, especially in the context of adult psychiatry, and call for the exclusion of adults with psychiatric disorders as sole underlying condition, from access to euthanasia.

**Five organisations and their proposed guidelines regarding the management of euthanasia in the context of psychiatry**

**I) The Organisation Brothers of Charity**

The congregation of the Brothers of Charity was founded in 1807 as the starting point for the development of a comprehensive mental health care network. Nowadays, the organisation of the Brothers of Charity provides mental health care in 13 psychiatric centres, 13 sheltered housing initiatives, and one centre for drug prevention and treatment. In March 2017, the organisation of the Brothers of Charity published its ‘Vision on euthanasia for psychological suffering in non-terminally ill patients' to be applied in its centres.

**II) The Belgian Advisory Committee on Bioethics**

The Belgian Advisory Committee on Bioethics was established by the Federal Government in 1993. It has 70 members from different disciplinary backgrounds, including a range of other characteristics reflecting the Belgian population's diversity. In September 2017, its “Opinion no. 73—Euthanasia in cases of non-terminally ill patients, psychological suffering and psychiatric disorders” was published

**III) The Flemish Association of Psychiatrists**

The Flemish Association of Psychiatrists was founded in 2004, with the aim to unite and represent all psychiatrists working in Flanders, to foster the quality of psychiatry as a mental health care specialism, and to inform the societal and political debate regarding psychiatric mental health issues. Close to 700 psychiatrists are associated members of the Flemish Association of Psychiatrists. In December 2017, the Association published its advisory text on “How to handle a euthanasia request in psychiatry in accordance with the legal due care criteria?”

**IV) Zorgnet-Icuro**

Zorgnet-Icuro was founded in 2016, with the aim to unite and represent all privately and publicly funded social profit health care organisations in Flanders. More than 775 health care organisations are associated members of Zorgnet-Icuro. In January 2018, its ethical advice on “End-of-life care for non-terminally ill patients with serious psychiatric disorders” was made public.

**V) Belgian Board of Physicians (Orde der Artsen)**

The Belgian Board of Physicians is an overarching institution that comprises all physicians (over 52,000) who practice medicine in Belgium, either temporarily or permanently. In April 2019, the Association published their deontological guideline a165002 on “the euthanasia practice concerning patients whose mental suffering results from a psychiatric disorder”.

**Court cases**

i) In 2015, the Belgian Federal Control and Evaluation Committee referred the first “euthanasia case, predominantly based on psychological suffering” to the Belgian Public Prosecutor as not all the legal requirements were deemed met. In 2019, the performing physician was dismissed of further legal proceedings as the Public Prosecutor concluded that the physician's acting was not “euthanasia” because the patient had drunk the provided lethal drugs herself, knowing it would immediately end her life. As the lethal dose was not injected by the physician, it was not considered “euthanasia” but “physician-assisted suicide.”
ii) In 2018, three Belgian physicians faced trial before a public jury, as they were accused of unlawful actions during the euthanasia assessment procedure and/or the act of euthanasia itself. In 2020, all three physicians were acquitted from the Belgian court of assise, although the performing physician may still face a correctional sentence.

The published guidelines also recommend a stronger involvement of an interdisciplinary team to enhance the quality of current psychiatric euthanasia assessment procedures. Furthermore, end-of-life consultation centers employ other types of health carers than physicians alone, i.e., psychologists, psychiatric nurses, and well-trained volunteers such as buddies. All these people may be involved in a patient's euthanasia procedure and may have an influential role in the euthanasia outcome. Recently, whereas buddy services were established to help these patients to cope with the euthanasia procedure that they may perceive as burdensome (15), rehabilitation-oriented support groups were established to help these patients with life-and-death considerations. All these health care professionals and volunteers may also have an unacknowledged but influential role in these euthanasia assessment procedures. Unfortunately, the concrete experiences and support needs of these carers have not yet been addressed in in-depth research endeavors.

Hence, the purpose of this research is (1) to explore health carers' experiences in their involvement in the management, assessment or other additional support of adult patients suffering predominantly from psychiatric conditions with a euthanasia request and (2) to explore their support needs in this regard.

## Methods

### Study Design

The semi-structured interview research design consisted of face-to-face interviews with health care professionals and volunteers in Flanders and Brussels, Belgium.

### Participants

All the participants were Dutch-speaking and had at least one concrete experience with euthanasia requests and procedures concerning adults with psychiatric conditions in the period 2016–2020. No further exclusion criteria were employed.

### Recruitment and Interview Procedure

Purposive sampling was used to ensure diversity and heterogeneity in terms of participants' affiliation with institutions holding different stances on “euthanasia and psychiatry” and being to a different extent confronted with these euthanasia procedures as regards the amount of experiences (sporadically vs. regularly) and the nature of the experiences (e.g., confronted with or engaged in euthanasia procedures that were still under review or that had been rejected, granted, performed or withdrawn).

Participants were recruited via assistance of our contact persons (see Box 2): (1) the end-of-life consultation center Vonkel; (2) the Organization Brothers of Charity; (3) REAKIRO in Louvain; and (4) the REBEL action group. The respective contact persons were asked to inform each associated potential participant about the interview study and to ask them to participate. Participants were also recruited via a notice on the sites, newsflashes and/or in the online newsletters of LEIF (Life End Information Forum), Recht op Waardig Sterven (the Flemish Right To Die with Dignity Society) and Flemish Association for Psychiatry.

Potential participants contacted MV, KC or the study assistant by phone or mail. The participants were then given an information letter and informed consent form that consisted of 2 main parts (see OSF). With the use of an interview topic guide (that can be found in our repository OSF repository) all interviews were conducted by MV, or a study assistant, both of whom have extensive experience in conducting interviews on end-of-life topics. Interviews were held at the participant's location of choice, except for 5 interviews which were held online by Whereby (16) due to the COVID-19 crisis lockdown regulations. Interviews lasted between 55 min and 2 h, and were audio recorded (the online interviews were recorded by Whereby's software and immediately transferred in an mp.3 format). Participants' time investment was compensated by means of a gift voucher.

### Data Management and Analysis

All interviews were transcribed verbatim by the two interviewers. After transcription, the audio files were kept under lock and key at Ghent University. The transcribed, anonymized data were stored on a secured Sync folder via encryption and transferred to QualiCoder (17), software for qualitative analysis. Only the interviewers, and co-authors KP and KC had access to the transcripts.

As our study was explorative, i.e., not based on any theoretical framework, MV, KP and KC used an open, thematic coding procedure, consisting of four phases; (1) identification and independent coding of all transcripts (MV), and the coding of 6 transcripts (3 by KC and 3 other transcripts by KP); (2) the substantive discussion on labeling and placing of the codes in subthemes (MV in close discussion with KC and KP); (3) the placing of these subthemes in overarching main themes (KC, MV); (4) the comparison and discussion of the findings, resulting in the coding structure (with all co-authors).

We used a model of sampling-based saturation, namely inductive thematic saturation (18), that relates to the emergence of new themes (defined as 7 consecutive interviews without new themes). We continued to recruit and conduct interviews so that the sample would be heterogenous in terms of socio-demographics, clinical profile, and clinical setting.

## Findings

The main characteristics of the 30 participants are listed in Table 1. The sample consisted of 16 physicians, 7 other care professionals (from psychiatric nurses to mobile support teams), and 7 volunteers, who engaged in one or more euthanasia procedures that were predominantly based on psychiatric conditions. Participating physicians held one or more roles regarding the handling of the euthanasia request:

- refused to discuss the request with the patient on principle grounds (*n* = 1);
- handled the clarification of euthanasia requests from one or more of their own patients themselves (attending physician) or referred one or more of their own patients to a colleague for further clarification (referring physician) (*n* = 7)
- were entrusted with the task to give one of the two legally required formal advices or an additional advice on the euthanasia request (advising physician) (*n* = 10);
- performed the act of euthanasia (*n* = 5);
- held a dissuasive stance against euthanasia in the context of psychiatry but were willing to explore and discuss the euthanasia request with the patient (*n* = 3).

**Table 1:** Healthcare Professionals and Volunteers' characteristics (*N* = 30).

**Characteristics**	**Medics^**a**^, *N* = 16**	**Care workers, *N* = 14**
Biological sex		
Male	11	7
Female	5	7
Age category		
<30 years	0	2
31–40 years	0	2
41–50 years	1	4
51–60 years	4	3
>61 years	11	3
Type of work environment^b^		
Private or group practice	5	0
Psychiatric units/psychiatric hospitals	7	2
Psychiatric care homes	2	3
Specialised end-of-life centres	5	5
Other	0	4
Number of concrete experiences in the year prior to the interview		
1–2 cases	1	3
3–5 cases	3	2
>5 cases	6	9
Specific role in euthanasia procedures^c^		
None	1	0
Attending/referring physician	7	0
Advising physician	10	0
Performing physician	1	0
Mobile teams	0	2
Psychiatric nurses	0	3
Experts by experience^d, e^	0	2
Spiritual carers^e^	0	3
Buddies^e^	0	3
(Secretary) consultants at end-of-life centres^e, f^	0	4

a *The following physicians were interviewed: 10 psychiatrists, 4 general practitioners and 2 other clinical specialists. The interviewed psychiatrists had expertise in, e.g., adult and old-age psychiatry, neuropsychiatry, forensic psychiatry, geriatric psychiatry, psychiatric substance abuse care*.

b *Some have more than one work environment*.

c *Some had experience in more than 1 role*.

d *Experts by experience, i.e., people classified with a (proneness to) mental illness, that are trained to provide support for someone who is “new” to the experience or entering rehabilitation approaches*.

e *Among these support team members, a variety of academic and professional background qualifications can be distinguished, e.g., former or present medics, psychologists, orthopedagogists, and communication scientists*.

f *These people are entrusted with e.g., the patient-intake and referral at end-of-life information or end-of-life consultation centers*.

The sample further consisted of 14 non-physicians, among them members holding one or more roles (see Table 1), e.g., mobile teams that provide psychiatric care and support in the patient's home setting (*n* = 2), psychiatric nurses working in a general hospital or in a psychiatric residential setting (*n* = 3), Experts by Experience, i.e., people with a history of mental distress who are trained to provide support for someone who is “new” to the experience (e.g., entering the euthanasia procedure and/or rehabilitation approaches, *n* = 2), buddies (*n* = 3) and spiritual carers (*n* = 3) entrusted with the task to assist, guide and/or support the patient throughout the euthanasia procedure, and consultants at end-of life information and/or consultation centers entrusted with patient intake (*n* = 5).

### Concrete Experiences With Euthanasia Procedures in the Context of Psychiatry

Participants' experiences, listed in Table 2, can be captured in eight overarching themes: their experiences regarding (1) the impact of euthanasia legislation and practice on the clinical trajectory, (2) impact on the therapeutic relationship, (3) the aspect of professional team collaboration, (4) the role and involvement of patients' relatives, (5) the collaboration with end-of-life centers (see Box 2), (6) the use of recently published guidelines, (7) the impact of recent court cases (see Box 2), and (8) their experiences with the act of euthanasia. In what follows, we discuss these themes in sequential order.

**Table 2:** Favorable and unfavorable experiences regarding euthanasia in the psychiatric context, reported by healthcare professionals and volunteers^a^.

**Theme**	**Favorable experiences**	**Unfavorable experiences**
Patients' clinical trajectory	*Benefits of a 2-track approach^b^* - Continuity of care, - Treatment non-abandonment, - Turning off ‘tunnel vision toward death' (C) - Exploration of rehabilitation options for patients (and relatives) - Empowerment (e.g., increased decision-making capacity and feelings of regaining some control in life) *Opening window of opportunity* - Serene in-depth discussion about death ideation unravels its underlying meaning (cry for help in dying vs. cry for extended aid) - The therapeutic effect of an exit-plan on patients' mindset: patients feel empowered to deal/cope with illness and other problems in life - Re-evaluation of diagnosis, treatment *Decreased suicidality* - Decreased suicidality	*Lack of or hampered 2-track approach* - Euthanasia request as reason for exclusion from ambulant treatment or residential stay - Rehabilitation low on options: understaffed, underfinanced - 2-track approach experienced as double-edged sword: whereas it may encourage some patients, it may discourage others *Closing window of opportunity* - The law itself inciting patients to fixate on death (discouraging/demoralising factor) *Suicidality persists* - No effect on suicidality
Relationship patient—physician/caregiver	- Meaningful care - Better ‘contextual' understanding of patients (C) - As patients feel heard, understood, respected, caregivers can reach a better connection/trusting relationship	- Difficulty to set personal/professional boundaries (C) - Difficulty to assume an appropriate role (due to inexperience/lack of training or tools)(C) - Therapeutic relationship treathened in case the euthanasia procedure is completely ‘outsourced' (C) - Insufficient (after)care for patients with euthanasia requests rejected/put on hold
Professional team collaboration	- Colleague intervision & support - Building up knowledge and expertise - Face & carry the responsibility, workload and/or emotional impact together	*Negativity bias* - Physicians willing to engage in the most cautious and careful manner face ‘stigma from colleagues' (P) *Experienced irregularities in the euthanasia assessment procedure* - No meaningful referral - Unmotivated advices, advice without conclusion *Poor management/follow-up* - Little or no time/space to discuss the case when ‘outsourced' (C) - Little or no intervision/supervision (C) - Little or no support (C)
Role and involvement of the patients' relatives	- Informing and involving relatives may result in mutual understanding, rehabilitation damaged/soured relationships - Heteroanamnesis = more contextual understanding, completion of “the puzzle” - Patients take time to prepare themselves and their loved ones for the end	- Relatives not or insufficiently consulted - No/little time/space for aftercare and closing
Collaboration with end-of-life information/consultation centres	- Offer low threshold for serene talks about death or for these patients whose euthanasia request are more often neglected/turned down by their treating physician - Highly needed and consulted 3rd line partner for the ‘individual professional' (support and assistance, expertise, independent partner, death track, objective assessment)	- Difficult collaboration with end-of-life consultation centres (different approach, ideological bias, etc.) or vice versa (poor physician administration/communication) - Lack of collaboration: treating physician/care team sidelined - Unprofessionalism: e.g., some volunteers not trained in the (para)medical field, patients as victim of internal rivalry - Overburdened: long waiting lists, understaffed
The use of guidelines	- Helpful in euthanasia assessment - Most concerns addressed by additional safeguards - Deontological guideline experienced as offering an authoritative framework	- Unhelpful (redundant, unpractical/vague, lacking in areas) - Flawed (biased, not uniform, discourages engagement, some paragraphs still unclear)
Impact of court cases	/	- Decreased willingness of physicians to engage - knock-on effect for patients with euthanasia request under review (concern request would no longer be assessed) - Missed opportunity for a more nuanced debate
Experiences during the performance of euthanasia	- Supportive moments shared with patient and relatives (gratitude, serene atmosphere)	- Patients' sudden change of mind (P) - Poor performance on a technical level (P)

a *(P) when the information was only mentioned by physicians and (C) when only mentioned by care workers*.

b *The 2-track approach is characterised by simultaneously focussing on the death track by means of exploring the patients' motives for requesting euthanasia and their eligibility for euthanasia on the one hand, while on the Life-track focussing on all alternatives to death, including rehabilitation options. This approach is recommended by the written guidelines and national Board of physician's deontology code on how to adequately manage euthanasia requests in the context of adult psychiatry*.

As regards the first theme, the impact on the “clinical trajectory,” most of the participants reported on the 2-track approach. This 2-track approach is recommended by the guidelines and characterized by simultaneously focusing on the *death-track* by means of the exploration of the reasons for and eligibility of a patient's euthanasia request, and on the *life-track* by means of the intensified exploration of rehabilitation and recovery options on a psychological, physical, social, and existential level, and psychiatric palliative care approaches.

Most participants experienced the 2-track approach as a positive challenge rather than a negative threat. Some of the participants experienced a lack or hampered 2-track approach on two levels: (1) colleagues not establishing a death track, e.g., when refusing to take euthanasia requests seriously or the reason for denying the patients' access to treatment, and/or (2) colleagues not following the life track in which reasonable alternatives to death were insufficiently or not explored, or insufficiently applied, e.g., the perceived remaining basic state-of-the art treatment options were ignored.

But something happened there, she went to (…) and she was put on a pedestal. A documentary was made of it, it was published in (newspaper), it was published in (popular magazine), so that created a certain, a certain something that left me, that left us, and what we had in mind, without a chance. And that was, that was terrible. Yes, and that has been a turning point, I think. I had the feeling that I wouldn't get another chance, or that we wouldn't get another chance. (…) Yes, and I would like to do that, but yes, I am but a nurse with experience, I would like to make an appeal: please, do not ever do this again, ever! Not with these people, certainly not with these people, with these kinds of problems. That should not be allowed. You are not allowed to do that. (Psychiatric nurse)

In addition, most interviewees reported on poor rehabilitation options available and insufficiently developed palliative care approaches that could focus more on comfort and holistic care needs than on the medical condition and curation in Belgium.

Some participants described a window of opportunities in the sense that the two-track approach in the euthanasia procedure may serve a 2-fold therapeutic objective. In their experience, acknowledging and validating the patient's difficulties in life and thoroughly discussing death ideation in a serene manner (without immediately initiating suicide protocols) may both appease the patient's mind and hence, decreases suicidality (by the prospect of a more dignified way of dying) and empower the patient to further explore the (underlying) meaning of the euthanasia request, to have their clinical trajectory re-evaluated or intensified. Other participants testified that euthanasia legislation closes this window of opportunity due to its discouraging and demoralizing effect. In their experience, the option of euthanasia had nudged some of their most vulnerable patients to apply for euthanasia, installed a tunnel vision toward death that discouraged them to give reasonable treatment options a fair chance of success and did not decrease suicidality.

Other participants had experiences with both scenarios and considered euthanasia legislation a double-edged sword: whereas it may encourage and empower a proportion of the patients to refocus on the *life track*, it may discourage or even further demoralize other patients, who feel less motivated to focus on the *life track* and are more swept into the *death track*.

Some participants partly ascribed this to the different motives for requesting euthanasia, be it a cry for additional help in life or a cry for help in dying.

“Puff, I think it's also a double-edged sword. On one hand, some people feel heard and get the idea of “well, now this may be a solution I can choose, isn't it? If it really…” There are many who literally say that, huh: “plan A is life-oriented, plan B, if I have it approved, then I feel supported, then I feel heard, that's a reason to work even harder on plan A.” But there are also people who will bury plan A much quicker, because they no longer have the courage to follow plan A. So, what I want to say is that it is a double-edged… well, that the outcome actually depends on the person herself. For one person it's, uhm, a solution to continue working on their treatment and to try to obtain a better quality of life, knowing that if they were to fail, they can be receiving euthanasia. And there are also those for whom it is just a lever to say, well, I choose not to do it anymore, uhm (Interviewer: So that it impedes potential treatments?). Impedes it, yes. I see it a little bit, well, you can compare it somewhat with, uh… There are suicides to life, and there are suicides to death. There are suicides that are clearly appeals to HELP ME, I want to live. There are also suicides that are not a cry for help, but definitely a cry for death. When you talk to the patients, you can get a clear idea, for example, of the suicides, the way they did the suicides, huh? There are some who will say, yes, well, I cut my arm, 4 months ago. And then I think, yes well, that's not a suicide attempt huh, that's, well, that's self-mutilation and that's actually a cry for help, not for death, but when they tell me, well, a year ago, I took 100 pills and I spent 5 days in intensive care, well, these people really want to die, don't they? They really want to die, don't they?” (Psychiatrist)

Some argued that these different motives were also seen in suicide attempt survivors, be it an acute cry for more attention and help in life or a passionate attempt to take revenge on others, vs. a more well-considered, rationalized road to death. Other participants argued that the debate regarding euthanasia on the one hand and suicide prevention on the other should not be mixed up, as they observed suicide ideation, attempts and deaths in both patients applying and not applying for euthanasia, as well as patients in both groups overcoming suicidality.

“So many things can change. A suicide, for example, can also be a signal. If it supposedly fails, you can notice afterwards, that that signal causes a lot of things that can actually lead to new equilibria and a meaningful balance. Likewise, the journey [*euthanasi*a *procedure*] can actually, due to all those selection criteria, indeed lead to things that result in something meaningful, and so on.” (General Physician)

According to some participants, the discussion on whether or not euthanasia could be considered a potential antidote for suicidality detracts the attention from the real question on the inherent lethality of psychiatric disorders, and suicide and euthanasia as different means to put an end on the long ordeal of suffering.

“Is death not immediately foreseeable with a psychiatric condition? That's the annoying thing, that you don't know that, isn't it? How many suicides do we have here? But well, I do have something against that, when we use euthanasia as a kind of antidote against, uh, against suicide, that's a totally different issue. But death and psychiatry, that is, why do we have all these governmental programmes against suicide, isn't that not dying of a psychiatric condition? Isn't that the second or third cause of death in young people? Is that not dying of a psychiatric condition? A psychiatric condition can be lethal. But we don't know when, right, that varies from one person to the next. We are left to make assessments all the time, how high is the risk of dying, huh, the risk of suicidality. And then that's about the lethality of some psychiatric conditions. If I remember correctly, the life expectancy for psychosis is 10 to 15 years lower than for other people, that's sad, isn't it? And then you have mortality, and you also have suffering. And many of the psychiatric people that I see [as advising physician], they suffer more than the average person with ALS who has to endure that for three years. 15 to 16 years of hospitalizations, no hospitalizations, I mean, you have these two factors, right? Lethality and suffering.” (Psychiatrist)

As regards the second theme, the impact of engaging in the euthanasia procedure on the relationship with the patient, participants phrased their involvement as an act of meaningful care. Notwithstanding the complexity and need surrounding these euthanasia assessment procedures, they experienced meaningful encounters and a deeper, more intimate, connection with patients, characterized by a sense of mutual respect and mutual understanding on the one hand and the greater intimacy and deeper knowledge of the individual behind the patient on the other due to the intensity of the euthanasia trajectory.

“It is a privilege for a physician to get very close to people in a very short space of time.” (General Physician)

All care workers valued the possibility to offer more holistic, individualized care for these patients that made it easier to help and support these patients in what each of them most needed support with. Although they all valued an even deeper and more contextual understanding of these patients, most of them also experienced its downside in terms of difficulties to set and maintain professional boundaries (e.g., over-involvement, the challenge of keeping a professional instead of a personal role).

Regarding professional team collaboration, the third theme, most participants experienced the added value of inter- and supervision (e.g., support & assistance, learning from other's experiences, to bear the emotional and professional load together). However, the following barriers were also reported, from physicians deliberately hindering the patient from starting the euthanasia assessment procedure by means of false information (e.g., telling the patient that the head of the hospital should give his approval for euthanasia assessment) or obstructing referral to a colleague or institution willing to engage in euthanasia assessment procedures, to physicians handling the euthanasia request with negligence or complacency due to poor communication (e.g., when not or insufficiently informing/consulting the patient's treating physician or caregivers), poor administration (e.g., providing insufficiently motivated formal advices) or contraventions (e.g., obtaining 1 instead of 2 legally required formal advices).

There was a lot of fuss going on about who would give the approval, and then the approvals were there, but they said, “if that person is giving approval, I no longer want to be involved.” So, actually the patient finds herself in a certain system that is playing above her head, that was not good, and so I took care of her but because of that situation, she went into a crisis, and I couldn't stand it, I couldn't stand the grief. It's very, very complicated, but of course when you see how it can affect a patient like that, that's just inhuman. Yes, and that's not right. No, no, absolutely not, no, no. So, it's actually a kind of internal conflict between, um, physicians that the patient has to suffer for? Yes and that's not okay. I was with her once when she was physically restrained, in isolation, which we hardly ever do anymore and I think that was one of the last times and even the last time that I saw that happening, a physically restrained patient, and the sadness was inhuman. (Psychiatric nurse)

Some of the interviewed psychiatric nurses pointed to the problem of euthanasia procedures that are “completely outsourced' to external organizations and therefore completely disconnected from the outpatient and/or residential treatment process. As a result, these psychiatric nurses experienced neither guidance nor support to provide appropriate care to these patients and their fellow patients, e.g., regarding the issue of “contagion,” especially among young adults, in terms of imitating the behavior and death ideation of fellow peers.

The involvement of patients' relatives during the euthanasia assessment procedure, the fourth theme, was experienced as an added value for (1) the physician involved as hetero-anamnesis offers a deeper understanding of the patient's personal, clinical and contextual history, present and future perspectives, (2) the relatives, as being recognized and not being side-lined may help them coping with the euthanasia procedure and—when euthanasia is performed—soften their mourning, (3) the patients, as they can shoulder along with the relatives in a joint trajectory, and (4) the patient-relative relationship as rehabilitation of soured relationships was reported. Some participants witnessed and criticized that patients' relatives were side-lined. Some physicians wanted to consult the relatives but felt unable to do so due to e.g., strong patient opposition, and/or felt unable to address the need for (after)care.

“When performing euthanasia, I usually say to those who are present, etc., you can always call me, and it may be necessary for, well, and you wouldn't be bothering me, and so on. Some people do call me, but not many. I myself don't take the initiative to take on another 4, 5, 6 people in grief counseling. I think that's the job of the general physician. I think that we indeed don't pay so much attention to that. Beforehand, yes, but after, no, I plead guilty. I don't do that well, I don't have the time and energy for that, I think, actually huh.” (…) “Yes, yes, yes, yes, there are already lawsuits, because among others [name physician] has already got a lawsuit about that. A patient who really said, ‘No I don't want it, I refuse that you inform [family members].' Yes okay, then you have to see. But also in the intervision, during the LEIF-physician training, we are told to try as much as possible and insist, and then you can witness very beautiful things occurring, of being able to say goodbye to those troubled relationships, because that is very important for the children, instead of being informed like, ‘hey, my father died, hey, by euthanasia and I knew nothing about it'. That's not easy, is it?” (General Physician).

In the experience of one physician, euthanasia requests were seldom based on patient's voluntary decision but most often due to pressure of relatives and as a consequence, the physician's duty is to muzzle the relatives' voices and strongly oppose euthanasia in this patient group.

“In a vast majority of cases, people are talked into the psychological suffering, which led the man in question to say under pressure from his family ‘alright, let's go for euthanasia then.' I know that because I knew him so well - well, I'm talking about different cases now – or her as well, whom I knew so well, that I knew this was actually not what she wanted. Because of their weakness, because of their illness, or because of their reduced resistance to go against them. Hence, only people who are so involved with their patients can judge that. Because you've known these people for forty years. And you know very well when they are telling the truth and when they are not. Then you know someone and say ‘all of a sudden their character has changed and all of a sudden they have made a request for euthanasia', that I wondered 'how can that be'? And then - but I think there are few people better able to judge than a general practitioner who knows his patients so well. I did feel that – well that was my impression at the end of my practice – that many people did not really want that. But, under pressure from the family- (Interviewer: Yes. And have you known cases or people whose request for euthanasia was genuine?) I don't think so. I don't think so.” (General Physician)

Whereas, the abovementioned findings resulted from the participants' experiences regarding the euthanasia legislation and -practice in general, the following experiences concern specific aspects that have changed the practice over the years. End-of-life information and consultation centers, the fifth theme, were praised as they offered a low threshold for serene talks about death and for patients whose euthanasia request are neglected or turned down by their treating physician. In addition, end-of-life consultation centers were experienced a highly needed and consulted 3rd line partner for the “individual professional.” However, participants working at these centers phrased that “this low threshold” is threatened by the difficulties to respond to the increasing imbalance between supply (when being understaffed and low in options for external referral) and demand (due to an increase in the number of patients applying for euthanasia and hence waiting list enrolment). Other participants reported an experienced lack of collaboration (when being side-lined) or poor professional collaboration with(in) end-of-life centers due to experienced unprofessionalism, e.g., some of the (peer-) volunteers not being trained in mental health care and/or patients being victim of internal rivalry between these centers.

As regards the role of written guidelines, the sixth theme, for some participants, they provide helpful guidance on translating and implementing the legal criteria in this patient group. The Belgian Board of Physicians' provision of a medical code of conduct recommending more stringent procedural criteria was experienced as “reassuring” to counter witnessed misuses (as physicians can be suspended). For others, this and other guidelines are deemed insufficiently helpful in terms of some passages being redundant, unpractical or vague (e.g., to what extent do physicians “have to take negative advices into account”) or lacking in areas (e.g., aftercare for patients with rejected requests is not addressed). Some criticized the existence of multiple guidelines as it jeopardizes uniformity. Some even consider these initiatives as discouraging physicians to engage in euthanasia assessment procedures as the additional criteria expand the workload (e.g., the recommendation of a roundtable discussion with all physicians involved).

Most participants referred to the negative impact of recent court cases, the seventh theme, in terms of (1) its factual dissuasive effect on (colleague-) physicians' engagement in euthanasia procedures and as a consequence, an increase of patients in already overburdened end-of-life consultation centers, (2) its devastating impact on patients with their request under review, as increased suicidality and even involuntary admission to a psychiatric ward had been reported. Some physicians involved would have suddenly imposed additional criteria going far beyond the ones stipulated in the guidelines (e.g., written agreement from relatives) or withdrawn from their engagement, (3) the legal uncertainty, e.g., on whether or not physician-assisted suicide is part of the law on euthanasia and hence, whether or not it should be reported to the Federal Evaluation- and Control Commission on Euthanasia, and (4) the missed opportunity for a more nuanced euthanasia debate as strong proponents and opponents were pitted against each other.

Finally, when a euthanasia procedure culminates in the performance of euthanasia, the eight theme, most participants reported that it happened in a serene atmosphere, in which the patient was surrounded by their relatives, who in turn expressed their gratitude to the participant involved. Some unfavorable experiences were also noted on a personal (e.g., the arm-needled patient's sudden change of mind), social (e.g., lack of serene atmosphere), and a practical-technical level.

“I'm going to tell you something, something terrible, well it was a terrible thing that happened to me, it was a young patient, ehm, with a problem of [names 2 mental disorders and describes the patient's physical appearance] inter alia, And well, this patient had obtained the needed advices and could convince me [to perform the euthanasia], and he, when looking at one of the attending parents with the needle in his arm, said: I would like to give it another try. (…) And I was taken aback.” (Physician)

### Support Needs

Eight support needs are distinguished and listed in Table 3. In what follows, we discuss these 8 themes, in sequential order. As regards theme 1, most of the participants, among whom well-trained physicians and nurses, plead for specific assessment approaches for the following specific patient groups: (1) patients with intellectual disabilities, (2) patients suffering from comorbid disorders and complex clinical pictures, (3) internees, (4) foreign patients, (5) young adults, and (6) “difficult patients,” e.g., the manipulative patient.

**Table 3:** Support needs as voiced by healthcare professionals and volunteers regarding euthanasia in the psychiatric context^a^.

**Topic**	**Reported support need**
Protocols for specific pathologies/sub-populations	Specific protocols for adequate assessment regarding the following sub-populations: - Patients with intellectual disabilities (P) - Patients with comorbid disorders/complex clinical pictures (P) - Internees (due to the specific environmental context) (P) - Foreign patients (due to e.g., lack of a juridical framework and the many administrative, practical, linguistic, cultural barriers) (P) - Young (<30 or at least <25) patients (C) - Guidance on how to deal with ‘difficult patients', e.g., the demanding, resistant, somatising, manipulative and aggravating patient (P)
Protocols specifically for non-physicians involved (C)	- Clear information on the euthanasia law and procedure - How to balance confidentiality/secrecy toward physicians (cf. suicide) - How to assume role as caregiver vs. friend (positioning toward the patient)
Implementation of the 2-track approach (P)	- Guidance and interpretation of the 2-track approach, e.g., Should these patients be obliged to continue treatment in the life-track, as this would violate the patient's right to refuse treatment? - Practical guidance on how to implement/find a balance in the 2-track approach to avoid tunnel vision toward death
(After)care for patients with psychiatric conditions	- More elaborated guidance on care/aftercare for patients with withdrawn euthanasia requests or with euthanasia request rejected
(After)care for caregivers (C)	- Organisational policies on improving, assisting and supporting the caregivers involved in more effective ways
Involvement of patient's social inner circle	- More practical and ethical guidance on their (extended) involvement, the viability/feasibility of involving the patient's relatives on who should be informed and the extent of their involvement in the euthanasia procedure
Education	- On the academic curriculum of all health care professionals: all EOLC options, including ‘euthanasia and psychiatry' - LEIF: more training hours (than 1.5 h) needed on ‘euthanasia and psychiatry', with emphasis on both the hitherto strict due process as well as the broad spectrum of rehabilitation - Specific training for Volunteers (C): role definition and responsibilities
Financial resources and staff	- More budget for mental health care >More incentives for proper palliative care for the mentally ill >More incentives for holistic therapeutic and rehabilitation approaches in psychiatry

a *(P) when the information was only mentioned by physicians and (C) when only mentioned by care workers*.

Whereas, the non-physicians reported needs for specific protocols to provide them and fellow colleagues with clear information on the euthanasia law and how to best deal with these euthanasia procedures (theme 2), some of the physicians and non-physicians pointed to the need of more practical guidance was needed on e.g., how to find a balance in the 2-track approach to avoid tunnel vision toward death (theme 3).

As regards theme 4, future updates of the guidelines for physicians need to cover areas that are still lacking, e.g., the (after)care for patients with euthanasia request rejected and withdrawn.

Moreover, some non-physicians proposed organizational policies improving, assisting, and supporting them e.g., to help them deal with own grief and emotions (theme 5). To date, the interviewed psychiatric nurses could only rely on suicide prevention policies within their walls. In the event of a suicide death within their walls, this can be discussed and borne *jointly* during team meetings. The opposite occurs if the euthanasia procedure is completely outsourced, precluding such team reflections and support, in turn inflicting an emotional toll on these care workers which in some cases caused these care workers to question their own competence. In such cases, these caregivers went through a difficult grieving process, inciting them to seek external professional help, to take a professional time-out or even to consider a new job.

And I started having doubts about my role as a care worker and so on, and well, that went reasonably well but then she died and I think a year ago, I went to see a psychologist to deal and cope with this, till now. Well, I've been through a lot with this young woman, right, with her attempts to hang herself and her destructive behavior, I am glad that I can admit to myself that it is okay to go to someone and to talk about it, to discuss it there for a while, and to process and digest it because yes, I do have a sort of “hangover” and I think that it will always feel like that. (…) But about your own wellbeing, uhm, within such a context, if you lose a patient to suicide, there's a procedure in place where you are allowed to see a psychologist or a psychiatric nurse for three sessions, for example. In the event of suicide, there is a team to which you can go to, but in the event of euthanasia there is no such team. (psychiatric nurse)

In addition, an ethical debate on the content and interpretation of the recommended 2-track approach and stronger relatives' involvement was deemed needed (theme 6). Examples cited by participants pertained to e.g., exploring the *life track* in light of patients' right to refuse treatment, or involving relatives while patients can legally enforce non-disclosure to relatives.

The need for more educational initiatives (theme 7) was expressed on the regular academic curriculum of all health care professionals (e.g., physicians, psychologists, nurses, social workers). LEIF training should include more training hours on euthanasia requests based on psychiatric conditions, with emphasis on both the hitherto strict due procedures as well as the broad spectrum of rehabilitation. Specific training for volunteers was deemed needed to help them to define their role and responsibilities and to set boundaries.

Finally, more budget for the underfinanced psychiatry is highly needed, including financial resources for proper palliative and rehabilitation approaches in psychiatry (theme 8).

Of course, palliative and rehabilitation approaches follow the same direction; they try to enhance the quality of life. You know, traditional psychiatric therapies are not always tailor-made. If you enter a psychiatric hospital, you must follow their programme, you have to go along with their programme, and if you don't go along with the programme, for example, then they tell you: “We don't think this treatment is something for you.” and you can go. That's why the importance of tailor-made care cannot be overestimated. That is our basic principle. There is nothing more exciting than to see what the best possible therapy programme is for each individual, and then to refine it along the treatment trajectory. The problem is that there are not enough resources and personnel to do more refining. There are successful therapy models, and I think a lot of so-called rehabilitation departments or recovery departments in traditional institutions are doing everything within their power. But the more traditional departments are not doing so very much. (Expert-by-Experience)

## Discussion

This in-depth interview study among health care professionals and volunteers aimed to explore their concrete experiences and support needs regarding the euthanasia trajectory in the context of adult psychiatry. Their concrete experiences were categorized in eight overarching themes and resulted in their reporting of eight support needs. We'll discuss the following 3 main findings:(1) the use of the guidelines and its recommended two-track approach, (2) the unfavorable experiences and urgent needs of non-medics, with an emphasis on the needs of those working in residential settings, and (3) the particular situation in Belgium following the euthanasia trials.

Our interview study followed a period in which multiple guidelines and a medical code of conduct were published, to allow these euthanasia cases to be dealt with adequately. Most of the participants experienced the guidelines helpful for euthanasia assessment but questioned whether the one-fits-all approach can be applied in the medical subdiscipline of psychiatry. They expressed the need to diversify for certain psychopathologies and subpopulations, e.g., the younger generation of patients. This issue seems even more relevant, since the recent study of the Dutch center of Expertise in Euthanasia revealed the trend of an increasing proportion of younger mentally ill requesting euthanasia (19). Also, in line with a previous article that made a critical point-by-point analysis of the guidelines (14), our findings confirm the value of and appreciation for the two-track approach as it may avoid the excesses of a narrowed focus on one single track. Nonetheless, our study found a need for more guidance on correct interpretation and proper implementation of the two-track approach. For instance, issues emerged on how to handle the tension between both tracks in the most effective manner, given the experience that exploring the euthanasia request may empower some patients but may discourage others to give alternatives for death a fair chance of success.

Only one Belgian previous study addressed psychiatric nurses' attitudes and experiences regarding the issue and showed that half of the responding psychiatric nurses had frequently been directly confronted (and 69% indirectly informed) with euthanasia requests predominantly based on psychiatric reasons (20). Our paper is the first to capture their experiences and needs more in-depth as well as those of many other mental health care workers who are underrepresented in research. As these people often spend more time with the patient (often also with the patient's most involved social inner circle) than physicians normally do, they can be considerably affected by these euthanasia trajectories. Even though all these mental health care workers appreciated the close(r) and deep(ened) relationship with the patient and considered their challenging work an act of meaningful care, most of them reported a lack of education and skills on this matter. In (residential) settings that ‘outsourced' euthanasia requests, these care workers faced distress that could exceed their own coping capacity, causing some to question their professional competence. In the event of these euthanasia cases being carried out, the care workers feeling side-lined during the euthanasia trajectory faced “disenfranchised grief,” grief when incurring a loss that is or cannot be openly acknowledged, validated, and mourned due to (perceived) social norms. Disenfranchised grief is not specific for the euthanasia practice in the context of adult psychiatry, as it is seen in health care workers, after being faced with patient deaths in a palliative care, suicide or COVID-19 mitigating context (21–26).

Third, and on a broader societal level, this interview study was conducted during a time of increased media attention and debate, following one euthanasia case where physicians stood criminal trial (see Box 2). Although increased attention and critical reflections are essential to identify shortcomings and to improve the practice, the Belgian practice seems to be confronted with a negative pendulum swing. None of the participants reported such events to be beneficial, as it complicated or even compromised a serene work atmosphere for physicians engaging in the euthanasia practice. This seems to have resulted in a growing reluctance to engage in euthanasia assessments, evidenced in a recent survey among experienced physicians (27). A similar trend was observed in The Netherlands (28, 29). Conversely, those who welcomed the practice being subjected to heavy scrutiny, expressed disappointment that it had not led to a thorough evaluation of euthanasia in adult psychiatry after the trial(s).

### Strengths and Limitations

This is the first in-depth study that uncovered the concrete experiences and support needs of a variety and relatively large sample of health care professionals and volunteers, with the inclusion of buddies, spiritual consultants, and expert by experience, who are specifically trained and/or experienced in supporting these patients during their euthanasia trajectory. We succeeded in providing a unique and representative sample of participants, varying in gender, age, work setting, expertise and concrete experiences in the euthanasia practice in the context of adult psychiatry. Our study has also some limitations. Selection bias may have occurred. For instance, there is evidence of the younger generation of psychiatrists being confronted with and (willing to be) engaged in euthanasia assessment procedures (11) but we did not succeed in holding interviews with them. Our sample of non-physicians did vary in age, but the sample of physicians did not, with most of the physicians older than 60. In addition, due to COVID 19-restrictions and potentially also due to the legal and emotional consequences regarding one high-profile euthanasia case being brought to court, a few planned interviews were postponed and ended up canceled. This led to the voices of e.g., psychologists working in residential psychiatric settings, to be missed. Also, only participants from Flanders, the Dutch-speaking region of Belgium, were included. Future research on this topic with health care professionals and volunteers in Wallonia, the French-speaking region, is recommended. As former research has pointed to profound cultural differences between both regions regarding e.g., knowledge on and attitudes toward euthanasia, and the (organization of the) euthanasia practice, different experiences and support needs can be assumed (30). Finally, this study's findings cannot be generalized to the situation in other countries with a legal framework on euthanasia, e.g., the Netherlands and Spain.

### Implications for Future Research, Policy, and Practice

As regards research, more insight is needed on the (dis)advantages of the two-track approach in terms of assets, premises, and potential pitfalls. Our study suggests that the outcome of this two-track approach may be related partially to patients' characteristics. It can also be related to the practical modalities of its implementation in the practice as well as to the feasibility of its implementation in diverse psychiatric settings. For instance, if and to what extent would it be beneficial for the patient (and fellow peers) to have the euthanasia request explored within and/or outside a residential setting? Why are such euthanasia requests outsourced? Why did some participants report being side-lined?

Also, given that these euthanasia trajectories and their outcomes affect so many actors directly or indirectly involved, future focus group studies bringing both the patient population and the health care team, including the patients social inner circle, together, may elucidate how and to what extent one can address and meet other actors' needs. Particularly the perspective of patients' social inner circle is missing, while a Dutch study found a considerable role in and impact of the euthanasia trajectory for them (31).

As regards practice and policy, the problem of “outsourcing” deserves the fullest attention. Not only because our findings reveal that the (understaffed) end-of-life consultation centers are overburdened with patients on growing waiting lists [the same trend is reported in The Netherlands (32)], but because this outsourcing may be in disagreement with the spirit of the euthanasia law or a shirking of medical responsibility. If the psychiatric nurses from a residential psychiatric setting are indeed side-lined concerning the euthanasia trajectory of an in-home patient, this may have been a violation of the law, that stipulates the consultation between the physician and (members of the) nursing team mandatory if the latter is in close contact with the patient (2). Also, more resources are needed for psychiatry to develop proper and sufficient rehabilitation, recovery and palliative care options to strengthen the health carers' capacity to effectively explore the *life track*. In addition, the guidelines' recommendation for strict procedural steps, e.g., the two-track approach, the involvement of all health carers in close contact to the patient, and the involvement of the patients' social inner circle needs to be elaborated in more detail, with respect to feasibility and risks involved, e.g., of violating patients' rights to confidentiality and privacy (33). Furthermore, our findings revealed that the handling of euthanasia requests require specific knowledge and a range of skills that are not (sufficiently) included in neither the existing academic curricula nor the existing training initiatives, e.g., managing a two-track approach, adequate communication and collaboration, and observing certain ethical tensions that arise. Last but certainly not least, euthanasia policies should also address the need to recognize, validate and address grief in the work context, to properly prevent and manage disenfranchised grief and related consequences, e.g., fatigue, burnout, and low-perceived work ability.

## Conclusion

This study yielded insight into the many positive and negative experiences of a variety of health care workers in dealing with euthanasia requests in adult psychiatry. They reported several support needs across the extensive euthanasia trajectory, pertaining to concrete management of thorny issues that guidelines do not (yet) touch on or only superficially. Suggestions to improve the euthanasia practice relate to tackling these existing issues, to enhancing education and training, to promoting incentives for psychiatric palliative and rehabilitation care approaches, and to paying sufficient attention to the impact of a euthanasia trajectory on all actors, including the patients and their social inner circle, involved.

## Data Availability Statement

The original contributions presented in the study are included in the article/supplementary material, further inquiries can be directed to the corresponding author/s.

## Ethics Statement

The studies involving human participants were reviewed and approved by the Medical Ethics Committee of the Brussels University Hospital with reference BUN 143201939499, from the Medical Ethics Committee of Ghent University Hospital with reference 2019/0456, and from the Medical Ethics Committee of the Brothers of Charity with reference OG054-2019-20. All interviewees were given an information letter and detailed informed consent. The interviews were held upon signature of the informed consent or upon verbal (recorded) agreement when the interviews had to take place online (due to COVID restrictions).

## Author Contributions

MV, KA, and KC were responsible for the study methodology and managed ethical approval. MV and a research assistant conducted the interviews under the supervision of KC and KA. MV, KP, and KC were responsible for the coding structures. All authors performed a critical review and revision of the coding structures and final manuscript.

## Funding

MV was funded by the Research Foundation Flanders via research project (G017818N) and PhD fellowship (1162618N).

## Conflict of Interest

MV has received research grants from the Research Foundation Flanders. The remaining authors declare that the research was conducted in the absence of any commercial or financial relationships that could be construed as a potential conflict of interest.

## Publisher's Note

All claims expressed in this article are solely those of the authors and do not necessarily represent those of their affiliated organizations, or those of the publisher, the editors and the reviewers. Any product that may be evaluated in this article, or claim that may be made by its manufacturer, is not guaranteed or endorsed by the publisher.
